# Supplementary material for: Zebrafish as a Human Muscle Model for Studying Age-Dependent Sarcopenia and Frailty
Source: Int J Mol Sci. 2024 Jun 3;25(11):6166. doi: 10.3390/ijms25116166 (PMC11172448; doi:10.3390/ijms25116166)
Supplement: Supplementary file 1 [file ijms-25-06166-s001.zip › ijms-3034363-supplementary.pdf]

Table S1. Frailty index

Parameter	2 mo		10 mo		30 mo		60 mo	
	mean ± SD	FI	mean ± SD	FI	mean ± SD	FI	mean ± SD	FI
Total distance (cm)	4667.74 ± 1386.53	0	5143.71 ± 1186.04	0	3164.34 ± 1420.14	0.25	2958.52 ± 723.49	0.25
Max speed (cm/s)	35.30 ± 11.63	0	31.96 ± 6.47	0	25.83 ± 5.79	0.25	18.55 ± 7.65	0.25
Mean speed (cm/s)	4.18 ± 1.02	0	4.61 ± 0.55	0	2.64 ± 1.18	0	2.46 ± 0.60	0.25
Muscle weight/body weight ratio	0.45 ± 0.04	0	0.48 ± 0.03	0	0.42 ± 0.08	0	0.39 ± 0.04	0.25
FI Mean		0		0		0.13 ± 0.14		0.25 ± 0.00

Reference values from zebrafish used to develop a FI.

Notes: values represent the mean ± SD for parameters measured from the 2 mo. 10 mo. 30 mo and 60 mo zebrafish (n = 8 animals/group). cm = centimeters; cm/s = centimeters/second.
